# Curcumin and/or omega-3 polyunsaturated fatty acids supplementation reduces insulin resistance and blood lipids in individuals with high risk of type 2 diabetes: a randomised controlled trial

**DOI:** 10.1186/s12944-019-0967-x

**Published:** 2019-01-26

**Authors:** Rohith N. Thota, Shamasunder H. Acharya, Manohar L. Garg

**Affiliations:** 1 Nutraceuticals Research Program, School of Biomedical Sciences & Pharmacy, University of Newcastle, 305C Medical Science Building, Callaghan, NSW 2308 Australia; 2 Department of Diabetes, John Hunter Hospital, Hunter New England Local Health District, New Lambton Heights, NSW Australia; 3 Riddet Institute, Massey University, Palmerston North, New Zealand

**Keywords:** Curcumin, Dyslipidaemia, Insulin sensitivity, Omega-3 polyunsaturated fatty acids, Randomised controlled trial

## Abstract

**Background:**

Lowering insulin resistance and dyslipidaemia may not only enhance glycaemic control but also preserve the β-cell function, reducing the overall risk of developing type 2 diabetes (T2D). The current study was aimed to evaluate the effects of curcumin and/or long-chain omega-3 polyunsaturated fatty acids (LCn-3PUFA) supplementation on glycaemic control and blood lipid levels in individuals at high risk of developing T2D.

**Methods:**

This was a 2 × 2 factorial, randomised, double-blinded, placebo-controlled study. Participants were allocated to either double placebo (PL) or curcumin plus placebo matching for LCn-3PUFA (CC), or LCn-3PUFA plus placebo matching for curcumin (FO), or curcumin plus LCn-3PUFA (CC-FO) for twelve weeks. Primary outcome of the trial was glycaemic indices (HbA1C, fasting glucose and insulin). Insulin resistance and sensitivity is measured using homeostatic model assessment model.

**Results:**

A total of sixty-four participants (PL, *n* = 16; CC, *n* = 15; FO, *n* = 17, CC-FO, n = 16) were included in the final analysis. Post-intervention, HbA1c and fasting glucose remained unchanged across all the groups. Insulin sensitivity was significantly improved in the CC supplemented group (32.7 ± 10.3%) compared to PL (*P* = 0.009). FO and CC-FO tended to improve insulin sensitivity by 14.6 ± 8.5% and 8.8 ± 7.7% respectively, but the difference did not reach significance. Triglyceride levels were further increased in the PL (26.9 ± 7.4%), however, CC and CC-FO supplementation reduced the triglycerides, FO resulted in the greatest reduction in triglycerides (− 16.4 ± 4.5%, *P* < 0.001).

**Conclusion:**

Reduction in insulin resistance and triglycerides by curcumin and LCn-3PUFA appears to be attractive strategies for lowering the risk of developing T2D. However, this study failed to demonstrate complimentary benefits of curcumin and LCn-3PUFA on glycaemic control.

**Trail registration:**

ACTRN12615000559516.

**Electronic supplementary material:**

The online version of this article (10.1186/s12944-019-0967-x) contains supplementary material, which is available to authorized users.

## Background

The period of transition from normal glucose tolerance to overt type 2 diabetes (T2D) is primarily mediated by Insulin resistance (IR), a marked independent predictor of progression to T2D in high risk (e.g. obese populations, pre-diabetics) individuals [1, 2]. The deficiency of insulin arising due to IR results in progressive deterioration of glucose homeostasis. Results from the British Whitehall II study indicated that IR commences several years prior to diabetes development indicating lowered insulin sensitivity (IS) and reduced β-cell function in the pre-diabetic stage [3]. IR is often associated with decreased clearance and increase in the hepatic secretion and of very low-density lipoproteins. As a result, levels of circulating TG are increased, a common feature observed in individuals with IR and metabolic syndrome [4]. IR, together with dyslipidaemia, represents a greater risk of developing both T2D and cardiovascular disease in high risk individuals [5]. Interventions targeting IR and dyslipidaemia might help delay the progression to disease state. Since T2D involves a multifactorial pathogenesis with longer duration, well-tolerated interventions that are cost effective with multiple mechanisms might provide a better solution for long term adherence to lower the risk of T2D.

Curcumin, a bioactive compound isolated from turmeric, has been shown to provide beneficial effects on IR and glucose intolerance in in-vitro and preclinical studies, [6, 7]. These effects were mediated via lowering of low grade inflammation via down-regulation of nuclear factor kappa-light-chain-enhancer of activated B cells (NF-kB) and cytoprotection of pancreatic β-cells by increasing the concentrations of anti-oxidant enzymes [6]. In mice models of diabetes, curcumin increased the expression of 5′ adenosine monophosphate-activated protein kinase (AMPK), a key regulator of glucose and lipid homeostasis [8, 9]. Clinical trials with curcumin supplementation [10–12] have shown promising results, therefore, further substantiation of the beneficial effects of curcumin on glycaemic control is warranted.

LCn-3PUFA (eicosapentaenoic acid, EPA; docosahexaenoic acid DHA) have been shown to be associated with lower incidence of diabetes [13]. LCn-3PUFA mediated increase in IS has been proposed to be a possible underlying mechanism for lowering the incidence of T2D [13]. In the preclinical studies, LCn-3PUFA increased IS via peroxisome-proliferator activated receptor-gamma (PPARγ) activation [14] that mediates the adipokines secretions, particularly adiponectin [15]; and by GPR 120 activation, that is involved in down regulating the pro-inflammatory pathways [16]. However, the evidence from the prospective studies, meta-analysis and randomised controlled trials has been contradictory, leading to ambiguity over beneficial effects of LCn-3PUFA [17, 18]. LCn-3PUFA have been known to reduce plasma triglycerides (TG), primarily through reducing hepatic very low-density lipoprotein (VLDL-TG) production, and to some extent from increasing the clearance of VLDL-TG [19]. As increased VLDL-TG production is an early manifestation of IR, LCn-3PUFA mediated reduction in VLDL-TG secretion might provide a complimentary beneficial effect on IR along with established triglyceride lowering effects in individuals at high risk and those with T2D. Since IR is a hallmark for progression of T2D, reducing IR might provide both beneficial effects on glycaemic control and preservation of beta cell function. Parallelly lowering the dyslipidaemia is of prime importance to alleviate the risk factors associated with IR. In this study we aimed to evaluate the effects of curcumin and/or LCn-3PUFA on glycaemic indices (HbA1c, fasting glucose and Insulin). In addition, the effects of supplementing curcumin and LCn-3PUFA on lipid and inflammatory markers were also examined.

## Methods

### Subjects

Participants with high risk of developing diabetes (assessed through Australian diabetes risk assessment tool, AUSDRISK) or with impaired fasting glucose (IFG) or impaired glucose tolerance (IGT), were recruited from the Hunter Region, New South Wales, Australia through recruitment flyers and media advertisements. They were screened through telephone interviews based on the inclusion criteria: age between 30 and 70 years; body mass index (BMI) must lie between 25 and 45 kg/m^2^; diagnosed either with IFG (fasting glucose 6.1–6.9 mmol/L), IGT (2-h plasma glucose ≥7.8 mmol/L - < 11.1 mmol/L) or both; HbA1c levels lie between 5.7–6.4% or they obtain a score of 12 or more in the AUSDRISK tool assessment (a non-invasive questionnaire to determine the risk of developing diabetes). Exclusion criteria included diagnosis with T2D; history of severe neurological diseases or seizures; gall bladder problems; pregnancy or planning to become pregnant or breastfeeding; on pacemakers; consuming > 2 serves of oily fish per week or take supplements known to influence blood glucose levels. All participants gave their written informed consent. This study was approved by the University of Newcastle and the Hunter New England Area Health Service Human Research Ethics Committees. The trial has been registered with Australia New Zealand Clinical Trial Registry (ACTRN12615000559516).

### Study design

This was a twelve-week 2 × 2 factorial, double-blinded, randomised controlled trial. Allocation of intervention was performed by a software-based (Random Allocation Software 1.0.0) randomisation technique, using alpha-numeric codes in blocks of 8. Participants who attended the baseline visit at Nutraceutical Research Program clinical trial facility or John Hunter hospital, were randomised to one of the four interventions: Placebo (PL, 2 x placebo tablets matching for curcumin plus 2x1000mg corn oil capsules per day), curcumin (CC, 2x500mg curcumin (Meriva®) tablets, providing 180 mg of curcumin plus 2x1000mg corn oil capsules per day), LCn-3PUFA (FO, 2x1000mg fish oil (EPAX 1050 TG) capsules providing 1.2 g DHA + EPA plus 2xplacebo tablets matching for curcumin) or double active (CC-FO, 2x500mg curcumin (Meriva®) tablets, providing 180 mg of curcumin plus 2x1000mg fish oil (EPAX 1050 TG) capsules providing 1.2 g DHA + EPA). Participants were advised to take two allocated tablets/capsules with morning and evening meals. They were also advised to maintain their routine dietary intake and physical activity during the study period. The compliance to the study interventions were measured during the follow-up and post-intervention visit using a capsule count and capsule intake log. Erythrocyte fatty acid analysis is also used to check the compliance for FO and CC-FO. Changes in the medications or any illness during the study duration were also recorded.

### Data collection and outcome measures

#### Primary and secondary outcomes

The primary outcome in this trial was to evaluate the effects of curcumin and/or LCn-3PUFA on parameters relating to glucose control i.e. glycosylated haemoglobin (HbA1c), fasting glucose, fasting insulin and IR. Secondary outcomes included lipid profile (total cholesterol, TC; TG; HDL-Cholesterol, HDL-C; LDL-Cholesterol, LDL-C; and Total: HDL-cholesterol ratio, TC: HDL-C), C-reactive protein (CRP) and whole blood cell count. Fasting (at-least for 10 h) blood samples were collected from participants during baseline and post-intervention visits into EDTA, fluoride/oxalate and serum clot + Gel + Clot activator vacutainer by a trained phlebotomist. HbA1c was measured through Bio-Rad Variant II HbA1c testing system by Hunter Area Pathology Service (HAPS), an accredited pathology laboratory for compliance with National Pathology Accreditation Advisory Council Standards. Fasting glucose, lipids, CRP and whole blood cell count was measured using VP autoanalyzer by HAPS. Homeostasis model assessment (HOMA) calculator (https://www.dtu.ox.ac.uk/homacalculator/) was used to estimate IR and IS (%S). Atherogenic index of plasma (AIP) values was derived by using formula log (TG: HDL-C). InsuTAG values were derived by using the formula fasting insulin x fasting triglycerides [20]. Erythrocyte fatty acids were determined using direct transesterification followed by gas chromatography (Hewlett Packard 7890A Series GC with Chemstations Version A.04.02) [21].

#### Anthropometry and body composition measurements

Body composition measurements (weight, percent body fat, muscle mass) were performed on baseline and post-intervention visit days using direct segmental multi-frequency bioelectrical impedance (InBody 230, Biospace Co., Ltd. Seoul, Korea) Height (cm) of the study participants was measured using a wall mounted roll up stadiometer (SE206, Seca). Waist circumference (cm) was measured using a tape measure positioned between about halfway between the bottom of the lowest rib and the top of hip bones, roughly in line with the belly button.

#### Questionnaires (diet, physical activity and medical history)

At baseline, medical history and demographic information was obtained from participants via self-administered questionnaire. Participants were asked to complete a three-day food diary before the baseline and 12-week visit days. These food diaries were analysed through FoodWorks, Xyris (version 8.0) to estimate the measure of participants’ habitual dietary intakes. Physical activity of the participants was assessed using the International Physical Activity Questionnaire (IPAQ) – long form version.

### Statistical analysis

Sample size was calculated using a computer program (PS Power and Sample Size Calculations Version 3.0), based on the previous data on mean changes in HbA1c (standard deviation of 0.5 units). Seventeen participants in each intervention group were required to give 80% power at 0.05 significance level for detection of 10% reduction in HbA1c. Accounting for drop-out rate of 20% we needed to recruit 20 participants for each treatment group. Data collected at the baseline was analysed for normality using histograms and Shapiro-Wilk’s test and presented as mean ± SEM (standard error of the mean) or median (IQR, interquartile range) as appropriate. Significant changes in the baseline data between the groups were assessed through analysis of variance (ANOVA) or Kruskal-Wallis when the normality assumption was not met. Post-intervention data was presented as mean ± SEM or median (IQR) of absolute change (post-intervention value minus baseline value) for log transformed values and blood cell count and as relative change [(absolute change/baseline value) * 100] for other variables. Changes from the base-line to post-intervention within-treatment group were assessed through paired t-test or Wilcoxon signed-rank test. Significant effects of intervention on mean changes in the variables between the groups were measured using two-way ANOVA with post hoc (tukey’s) analysis. Correlations between the independent variables and dependent variables were obtained using Pearson product-moment correlation coefficient analysis. Based on the significant correlation values, Analysis of covariance (ANCOVA) was performed on an optimal set of confounding variables.

## Results

### Baseline characteristics

**Fig. 1:**
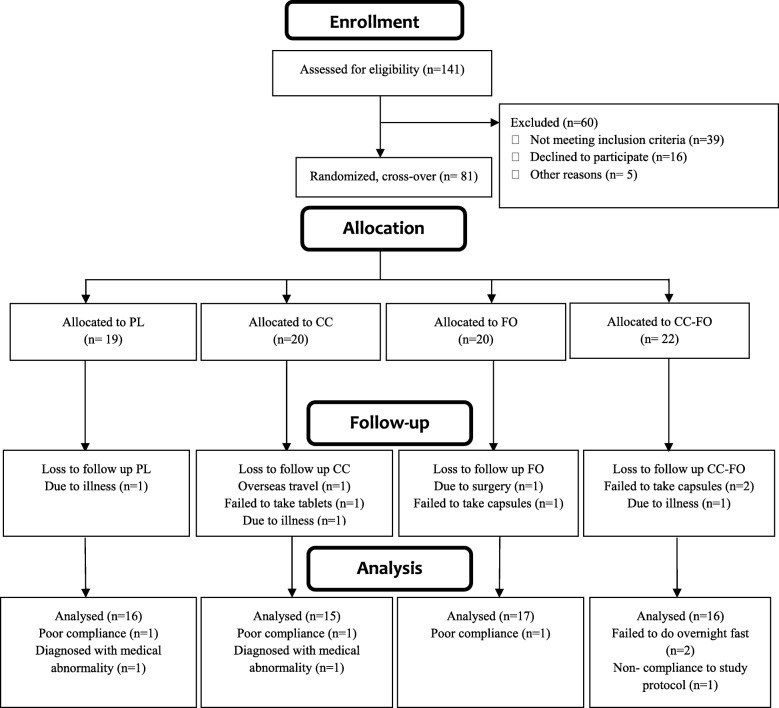
Consolidated Standards of Reporting Trials (CONSORT) flow chart– trial protocol

Eighty-one participants were randomised in this study. Number of participants allocated to the individual intervention groups and drop-outs are presented in the Consolidated Standards of Reporting Trials (CONSORT) flow chart (Fig. 1). There were no statistically significant differences observed in all the baseline characteristics between the four groups (Tables 1 and 2).

**Table 1:** Baseline general characteristics of the trial participants

CHARACTERISTICS	Total (*n* = 64)	PL (*n* = 16)	CC (*n* = 15)	FO (*n* = 17)	CC-FO (*n* = 16)	*P*-VALUE
Age (years)	55 ± 1.3	50 ± 2.5	55 ± 2.8	58 ± 2.5	57 ± 2.2	0.106
Males/females (n/n)	26/38	7/9	6/9	7/10	6/10	–
Ethnicity – no (%)						
Caucasian	50 (78.12)	12 (75)	12 (80)	13 (76.5)	13 (81)	–
Asian	7 (10.93)	2 (12.5)	2 (13.3)	1 (5.8)	2 (12.5)	–
Others	7 (10.93)	2 (12.5)	1 (6.6)	3 (17.6)	1 (6.25)	–
Anthropometry measures						
Body weight (kg)	88.5 ± 2.1	91.0 ± 4.6	88.1 ± 3.5	85.7 ± 4.9	89.9 ± 3.33	0.266
Muscle mass (kg)	32.4 ± 1.2	32.7 ± 1.2	34.2 ± 2.3	32.6 ± 3.6	30.3 ± 1.5	0.432
Body mass index (kg. m^−2^)	31.1 ± 0.7	31.9 ± 1.7	30.9 ± 1.2	30 ± 1.4	31.7 ± 1.2	0.468
Waist circumference(cm)	104.7 ± 1.5	105.7 ± 3.6	104.9 ± 2.7	102.5 ± 3.4	105.8 ± 1.9	0.086
Waist-hip ratio	0.96 ± 0.01	0.97 ± 0.01	0.96 ± 0.01	0.96 ± 0.06	0.99 ± 0.01	0.568
Percent body fat (%)	36.1 ± 1.3	35.3 ± 2.2	34.8 ± 2.5	35.3 ± 2.7	39.2 ± 2.4	0.507
Family history						
Family history of diabetes – no. (%)	31 (48.4)	8 (50)	11 (73.3)	4 (23.52)	8 (50)	–
Family history of cholesterol - no (%)	32 (50)	9 (56.25)	8 (53.33)	8 (47.05)	7 (43.75)	–
Family history of blood pressure – no (%)	13 (81.25)	13 (81.25)	9 (60)	12 (70.5)	10 (62.5)	–
Alcohol intake – no. (%)	54 (78.1)	10 (62.5)	15 (100.0)	14 (82.3)	15 (100.0)	–
Smoking – no. (%)	5 (7.8)	3 (18.7)	1 (6.6)	0 (0.0)	1 (6.3)	–
MEDICATION AND SUPPLEMENT USE						
Anti-hypertensives no. (%)	19 (29.68)	3 (18.75)	6 (40)	7 (41.17)	3 (18.75)	–
Anti-depressants no. (%)	10 (16.6)	6 (37.5)	3 (20)	0 (0)	1 (6.25)	–
Cholesterol lowering medications no. (%)	10 (16.6)	1 (6.25)	3 (20)	4 (23.52)	2 (12.5)	–
Antacids no. (%)	9 (14.06)	2 (12.5)	4 (26.67)	3 (17.6)	0 (0)	–
Supplements no. (%)	32 (50)	7 (43.75)	11 (73.3)	8 (47.05)	6 (37.5)	–

Data is presented as mean ± SEM or median (IQR), unless otherwise specified. *n* number of participants, *PL* double placebo, *CC* curcumin, *FO* Fish oil, *CC-FO* curcumin plus fish oil, *Kg* kilogram, *cm* centimetre, % percent, *Kj* Kilojoule, *METs* Metabolic equivalents, *SEM* Standard error of the mean, *IQR* interquartile range

**Table 2:** Baseline blood parameters of the trial participants

CHARACTERISTICS	Total (*n* = 64)	PL (*n* = 16)	CC (*n* = 15)	FO (*n* = 17)	CC-FO (*n* = 16)	*P*-VALUE
Glycaemic control parameters						
Fasting plasma glucose (mmol/l)	5.6 ± 0.1	5.2 ± 0.1	5.7 ± 0.2	5.7 ± 0.1	5.8 ± 0.2	0.168
Fasting serum insulin (mIU/L)	11.5 ± 0.8	11.5 ± 1.4	10.9 ± 1.5	12.6 ± 2.2	11.0 ± 1.5	0.957
HOMA2 IR	1.3 (0.9)	1.4 (1)	1.3 (0.6)	1.3 (0.9)	1.3 (1.3)	0.977
HOMA2 %S	76.1 (53.4)	73.7 (53.7)	79.4 (37.9)	76.6 (53.2)	78.4 (75.6)	0.975
InsuTAG	78.4 (89.5)	111.5 (119.6)	78.5 (85.0)	100.4 (103.7)	72.4 (57.6)	0.455
HbA1c (%)	5.5 ± 0.04	5.3 ± 0.1	5.5 ± 0.1	5.6 ± 0.1	5.6 ± 0.1	0.207
Lipid parameters						
Total cholesterol (mmol/L)	5.6 ± 0.1	5.7 ± 0.4	5.4 ± 0.3	5.7 ± 0.2	5.4 ± 0.2	0.795
LDL-C (mmol/L)	3.6 ± 0.1	3.8 ± 0.3	3.5 ± 0.2	3.7 ± 0.2	3.4 ± 0.2	0.583
HDL-C (mmol/L)	1.4 ± 0.0	1.3 ± 0.1	1.4 ± 0.1	1.4 ± 0.1	1.5 ± 0.1	0.195
Triglycerides (mmol/L)	1.4 ± 0.1	1.7 ± 0.2	1.4 ± 0.1	1.4 ± 0.1	1.2 ± 0.1	0.170
Total: HDL-C	4.2 (1.6)	4.5 (2.1)	4.3 (2.0)	4.1 (0.9)	3.8 (1.4)	0.120
Inflammation and blood cell count						
CRP (mg/L)	2 (3)	2.3 (4.7)	1.7 (2)	2.5 (3.4)	1.3 (2.4)	0.250
White blood cells (10^9^/L)	6.8 ± 0.2	7.0 ± 0.5	7.0 ± 0.5	7.0 ± 0.3	6.3 ± 0.5	0.680
Neutrophils (10^9^/L)	3.9 (1.5)	4.0 (1.7)	4.3 (2.2)	3.9 (0.8)	3.6 (2.0)	0.579
Lymphocytes (10^9^/L)	1.9 ± 0.9	2.0 ± 0.7	2.1 ± 0.2	2.3 ± 0.2	1.8 ± 0.9	0.241
Monocytes (10^9^/L)	0.5 (0.2)	0.4 (0.3)	0.5 (0.2)	0.5 (0.3)	0.5 (0.2)	0.203
Platelets (10^9^/L)	229 (73.5)	223 (46)	232 (74)	243 (104)	241 (77)	0.893

Data reported as means±SEM. or median (IQR). *PL* double placebo, *C* curcumin, *FO* fish oil, *CC-FO* curcumin + fish oil, *HOMA2 IR* homeostatic model assessment for insulin resistance, *HOMA2 %S* insulin sensitivity, *HbA1c* glycosylated haemoglobin, *CRP* C-reactive protein, *LDL-C* LDL-Cholesterol, *HDL-C* HDL-cholesterol, *Total: HDL-C* Total cholesterol to HDL-C ratio

### Body composition

Comparisons between the groups showed no significant differences observed between the mean changes in body composition measurements (body weight, muscle mass, BMI, percent body fat, waist circumference (data not presented) with all the three interventions.

### Glycaemic indices

**Fig. 2:**
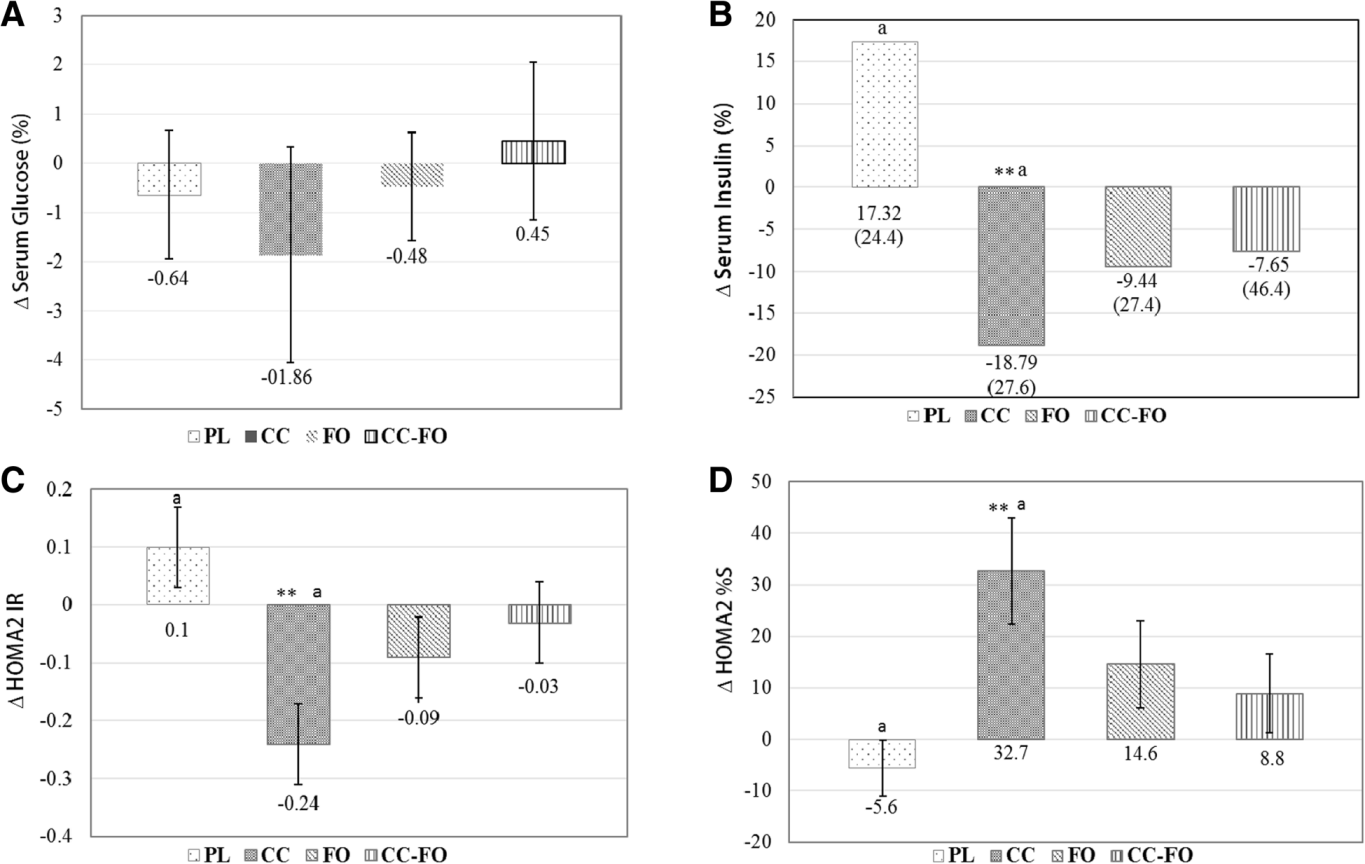
Changes in the outcome measures from baseline to post intervention with-in and between double placebo (PL), curcumin (CC), fish oil (FO) and curcumin + fish oil (CC-FO); **a** Change in the serum glucose (%); **b** Change in the serum insulin (%). **c** Change in homeostatic model assessment for insulin resistance (log transformed) (HOMA2 IR); **d** Change in insulin sensitivity. Data presented as mean ± SEM or median (IQR) as appropriate. Significant changes from baseline indicated by * *p* < 0.05, ** *p* < 0.01. Means with same lower letter indicate significant differences between the groups

After 12 weeks of intervention with curcumin and/or LCn-3PUFA, no significant changes were observed in fasting glucose (Fig. 2a) and HbA1c (Table 3). Fasting insulin was significantly (*P* = 0.005) reduced only in CC group [− 18.79 (27.6)%] from baseline (Fig. 2b). When compared between the groups, CC lowered the fasting insulin significantly (*P* = 0.002), but not FO (*P* = 0.054) and CC-FO (*P* = 0.101) compared with PL group (Fig. 2b). Similar trends were observed in the changes with HOMA2 IR (Fig. 2c) and HOMA-S (Fig. 2d) within curcumin treatment group and between CC and PL group.

**Table 3:** Changes in the outcome measures from baseline to post-intervention

CHARACTERISTICS	PL (*n* = 16)	CC (*n* = 15)	FO (*n* = 17)	CC-FO (*n* = 16)	*P*-VALUE
Glycaemic control parameters					
Fasting plasma glucose (%)	−0.6 ± 1.3	−1.9 ± 2.2	−0.5 ± 1.1	0.5 ± 1.6	0.851
HbA1c (%)	1.7 ± 0.6*	1.0 ± 0.7	2.0 ± 0.8*	2.2 ± 1.2	0.765
Fructosamine (umol/L)	2.2 (14.7)	0.8 (13.3)	2.7 (9.1)	1.7 (10.6)	0.776
lipid parameters (%)					
Total cholesterol (mmol/L)	0 (3.7)	1.92 (14.4)	0 (10.8)	2.7 (19.2)	0.767
LDL-C (mmol/L)	−2.7 (10.2)	−1.6 (25.1)	3.0 (17.9)	6.5 (29.7)	0.120
HDL-C (mmol/L)	0 (15.3)	0 (15.8)	6.3 (12.5)	0 (19.0)	0.333
Total: HDL-C	3.3 (12.4)	−5.2 (9.7)	−2.6 (16.5)	2.6 (12.2)	0.180
Inflammation and blood cell count					
CRP (mg/L)	−0.3 (1.2)	0.3 (0.6)	0.3 (1.4)	0 (0.9)	0.080
White blood cells (10^9^/L)	0.2 (1.1)	−0.7 (1.3)*	−0.4 (0.8)*	− 0.4 (1.2)	0.339
Neutrophils (10^9^/L)	0.2 (0.8)	−0.1 (0.8)	−0.5 (0.9)	− 0.3 (1.1)	0.070
Lymphocytes (10^9^/L)	0.04 ± 0.1	−0.3 ± 0.1*	0.1 ± 0.1	0.1 ± 0.1	0.024
Monocytes (10^9^/L)	−0.01 ± 0.0	−0.1 ± 0.0**	0.0 ± 0.0	−0.01 ± 0.0	0.290
Platelets (10^9^/L)	3 (27)	2 (19)	2 (29)	−10 (24)	0.618

Data presented as mean ± SEM or median (IQR) as appropriate. Significant change from baseline, *p < 0.05, **p < 0.01 represents the significant differences from the baseline with-in treatment groups. *PL* placebo, *CC* curcumin, *FO* fish oil, *CC-FO* curcumin + fish oil, *HbA1c* glycated haemoglobin, *LDL-C* LDL-cholesterol, *HDL* HDL-cholesterol, *Total: HDL* total cholesterol-to-HDL ratio, *CRP* C-reactive protein

### Blood lipids

**Fig. 3:**
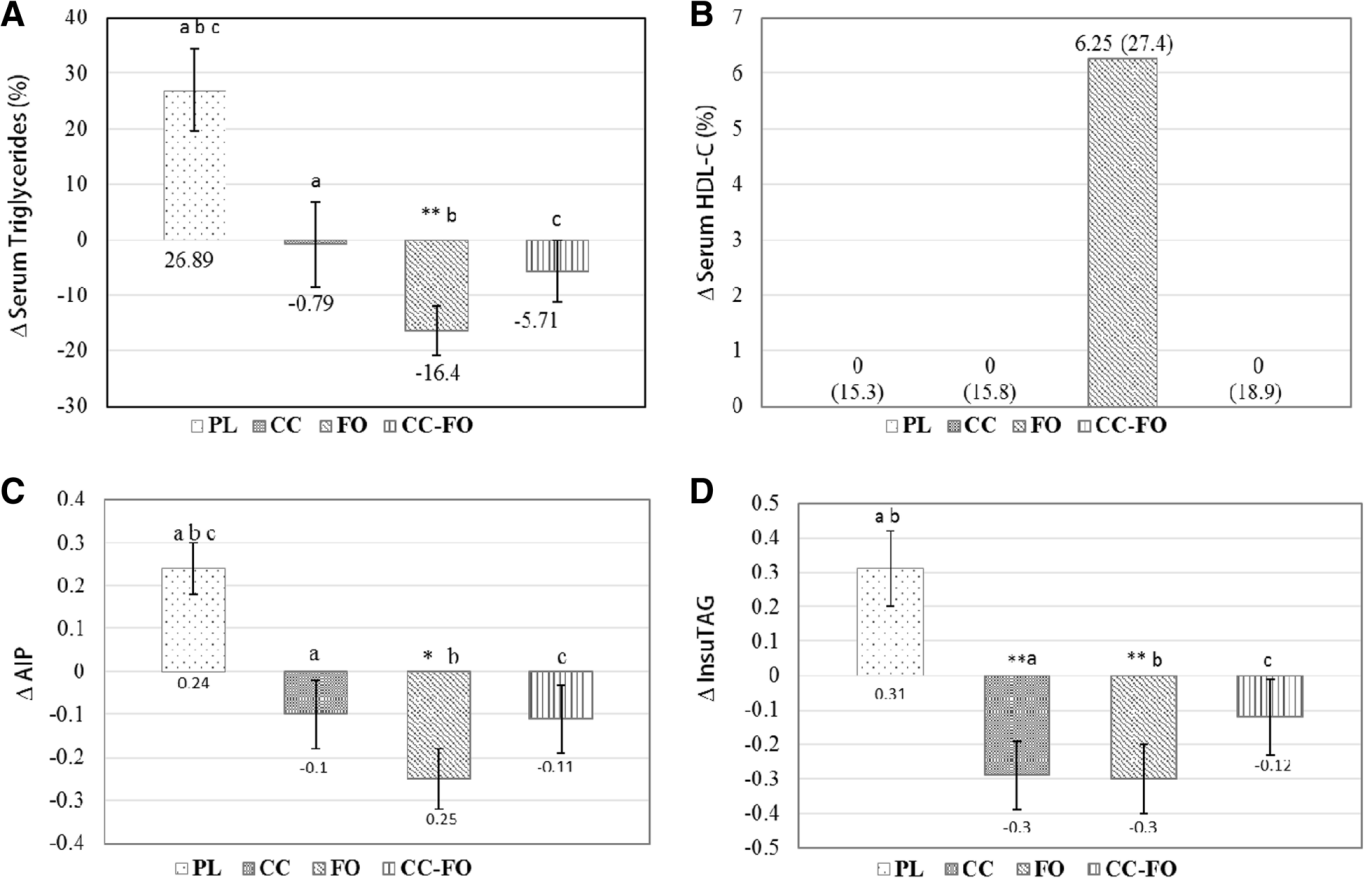
Changes in the outcome measures from baseline to post intervention and between double placebo (PL), curcumin (CC), fish oil (FO) and curcumin + fish oil (CC-FO); **a** Change in the serum triglyceride (%). **b** Change in the serum HDL-Cholesterol (%); **c** Change in atherogenic index of plasma (AIP) **d**. Change in InsuTAG (log transformed). Data presented as mean ± SEM or median (IQR) as appropriate. Significant changes from baseline indicated by * *p* < 0.05, ** *p* < 0.01. Means with same lower letter indicate significant differences between the groups

Post intervention, TG levels were significantly (*P* = 0.007) reduced (− 16.54 ± 4.5%) in FO group and increased (26.8 ± 7.4%, P = 0.007) in PL group (Fig. 3a). However, when compared between the PL group (0.24, *P* = 0.001), reduction in TG was significant with FO (*P* = 0.000), CC (*P* = 0.019) and CC-FO (*P* = 0.003) (Fig. 3a). Similar trends observed with absolute change in AIP with FO (− 0.25, *P* = 0.004) and PL groups (Fig. 3b). AIP was significantly reduced in FO (*P* = 0.000), CC(*P* = 0.025), and CC-FO(*P* = 0.008) groups compared with PL group. There were no statistically significant differences observed in TC (Table 3), HDL-C (Fig. 3c), LDL-C and TC: HDL-C between the groups (Table 3). Both CC and FO exhibited similar magnitude of reductions in absolute change of InsuTAG by − 0.29 (0.013) and − 0.3 (0.009) respectively from baseline (Fig. 3d). When compared between the groups, significant reductions in InsuTAG were observed with supplementation of CC (0.001), FO (0.001) and CC-FO (0.030) compared to PL.

### Inflammation and blood cell count

Both CC and FO and CC-FO did not exhibit any significant effects on CRP in with-in group or between the group analysis (Table 3). WBC counts were significantly reduced in the CC and FO group from the baseline (Table 3). However, this trend on white cells count by CC and FO were not seen in the between-group analysis when compared with PL (Table 3).

### Dietary intake, physical activity, compliance and adverse effects

Comparisons between the groups showed no significant differences, both baseline and post-intervention, on mean diet intake and physical activity levels of the participants (Additional file 1: Table S1). Post intervention, mean changes in body composition measurements (body weight, muscle mass, BMI, percent body fat, waist circumference) were also not changed in the participants (data not presented). The mean compliance of the participants to the study products was 94.86 ± 5.8% (capsule count). Erythrocyte fatty acid content of DHA and EPA were significantly increased by FO (EPA 29.3 (59.5) %; DHA 54.0 (43.6) %) and CC-FO (EPA 27.8 (61.8) %; DHA 39.6 (55.9) %). CC and FO were well tolerated by the participants and no adverse events were reported during the study period. ANCOVA analysis indicated physical activity as potential confounder for relative change in the fasting insulin levels (*P* = 0.026) and fasting triglycerides (*P* = 0.013), but the effect of CC (*P* = 0.012) for fasting insulin and FO (0.018) remained after adjusting for physical activity.

## Discussion

In attempts to find well-tolerated candidates with potential to lower the risk or to the delay the progression of T2D, we evaluated curcumin and/or LCn-3PUFA for achieving the glycaemic control in individuals at high risk of developing T2D. The results presented demonstrate that supplementation with curcumin is effective in lowering the serum insulin levels along with improvement in IS. Curcumin also showed a significant reduction in the serum TG and AIP. However, the magnitude of reduction for the serum triglycerides and AIP was higher in the individuals receiving fish oil. The effects in participants receiving both curcumin and LCn-3PUFA were in line with the individual supplementation groups, however we did not find any additive or complimentary benefits. Despite improving the risk factors (such as IR) by curcumin and LCn-3PUFA, there were no immediate beneficial effects observed on glycaemic control in 12 weeks.

In preclinical studies with diet-induced IR models, curcumin mediated amelioration of IR was primarily through decreased NFκB activity, increased anti-oxidant transcription factors (Foxo1) and activation of AMPK α-subunit by phosphorylating Thr-172 residue, thereby increasing the IS and overall glucose control [6, 22]. In the current study, curcumin effectively lowered the fasting insulin levels and IR (HOMA2 IR) and improved the IS (evaluated through HOMA2 %S), in line with reports from the other clinical trials [10, 11]. We have previously demonstrated that acute supplementation with same dose of curcumin significantly lowered the meal stimulated postprandial insulin response (AUC) by 26% [23] compared with placebo. Controlling postprandial insulin responses could be one of the potential underlying mechanisms for reduction in the elevated fasting concentrations of insulin by curcumin. In contrast to the glucose lowering reports from other previously published studies [10, 11] with longer duration of supplementation, in this study there was no significant effects of curcumin on both fasting glucose and HbA1c. This controversy may be explained by the fact that fasting blood glucose levels were only marginally higher in the current study and that 12 weeks may be inadequate to influence changes in HbA1c. Moreover, the dose and formulation of the curcumin employed in these trials were completely different.

The effects of curcumin on blood lipids are still unclear [24, 25]. Recent publication from our lab on randomised controlled trial with curcumin supplementation reported enhancement of the cholesterol lowering properties of phytosterols [26]. In the current study, curcumin did not influence cholesterol levels, however it prevented the rise in serum triglycerides that was otherwise evident in the placebo group. The concentration of fasting serum insulin was positively correlated with serum triglycerides (r = 0.274, *P* = 0.02). Curcumin supplementation blocked the rise in triglycerides, which could probably be mediated via reduction in the insulin levels. AIP, an independent indicator of coronary artery disease and atherosclerosis [27–29] was significantly lowered by curcumin compared to the placebo group, in line with a randomised controlled trial on beneficial effects of curcumin on atherogenic risks [30].

Several clinical trials and meta-analysis have confirmed triglyceride lowering effects of LCn-3PUFA [31, 32]. In the current study, LCn-3PUFA supplementation effectively reduced the circulating triglycerides and AIP. Significant elevation in the erythrocyte levels of both EPA and DHA in the FO receiving groups also confirms its LCn-3PUFA mediated triglyceride lowering effects. On the other hand, a trend was observed for improvements in insulin sensitivity (*p* = 0.054) with FO supplementation. These observations on beneficial effects of LCn-3PUFA for improving IS and glucose intolerance is in support with the existing study reports [33–35]. A meta-analysis from our research group has previously reported that trials with supplementation of FO ≥6 weeks, showed a significant improvement in IR in women, but not in men [35]. This sex dependent effect could partly be one of the key reasons that could be affecting overall outcomes on IS with FO supplementation. Improving omega-3 index (sum of EPA and DHA in erythrocytes) has been associated with increased IS and lower incidence of diabetes [36]. FO supplementation improved the omega-3 index of participants to a significant extent, suggesting a favourable metabolic outcome. One of the key mechanism of LCn-3PUFA may be via resolution of inflammation, which plays a key role in resolving persistent tissue low-grade inflammation in chronic metabolic diseases [37, 38]. This is believed to be mediated through changes in the membrane phospholipid composition of the cells participating in inflammation process. Studies in humans with doses ranging from (1.3 g to 2.5 g per day) have shown decreased production of arachidonic acid, a precursor for pro-inflammatory eicosanoids [39]. In the current study, with 1.3 g EPA + DHA per day, we have shown significant reduction in the erythrocyte levels of arachidonic acid, indicating reduced production of pro-inflammatory molecules. As CRP is more reflective of any acute inflammation or even an infection [40] than resolution of inflammation, we could not find any changes in CRP with FO supplementation in this study.

We have recently introduced a novel marker with high sensitivity and specificity values, InsuTAG, that considers both insulin and triglycerides for identifying individuals with IR and metabolic syndrome [24]. Supplementation of curcumin significantly reduced the InsuTAG levels in the current study, which could primarily be a result from reduction in the fasting insulin. LCn-3PUFA supplementation also shown similar significant reduction in the InsuTAG levels. LCn-3PUFA mediated reduction in InsuTAG could be mainly from lowering the plasma triglycerides. Though the effects on IR are mediated through different mechanism by curcumin and LCn-3PUFA, overall reduction in the InsuTAG implies potential attractive strategy for lowering the multiple risk factors for cardio-metabolic disorders.

Recruitment of participants with high risk of developing diabetes, we used blood glucose levels and AUSDRISK questionnaire to determine the risk of diabetes. Subgroup analysis indicated heterogeneity in study population, with more than half the number of the participants having fasting blood glucose levels > 5.5 mmol/L. Fasting plasma glucose was significantly lowered from baseline with LCn-3PUFA (*P* = 0.008) supplementation in participants with baseline blood glucose level > 5.5. Also, there was a clear trend in beneficial effects of LCn-3PUFA on fasting serum inuslin (Additional file 1: Figure S1B) in participants with high baseline blood glucose levels, indicating LCn-3PUFA supplementation could be of more beneficial in people with IFG than normal glucose levels. On the other hand, there was no significant difference in the effects of curcumin on these parameters between the two groups, except a significant reduction in the fasting insulin from baseline in participants with baseline blood glucose levels > 5.5 mmol/L. Moreover, we have not particularly stratified the number of participants in groups based on sex. To examine the treatment effects on relative changes in glucose and lipid variables separately in males and females has led to interesting observations. The mean changes in fasting insulin, fasting triglycerides, HDL-C and HOMA2(%S) with FO and curcumin were distinctively different in males and females, with supplementation greatly favouring males (Additional file 1: Table S2). One of the reasons for these differences could be slightly higher (but not significant) baseline levels of the above parameters in men than women (except for HDL-C). Together these sub-group analysis reports indicate gender stratification and a study population with high baseline glucose status is necessary to further substantiate the beneficial effects of curcumin and LCn-3PUFA in lowering the risk of T2D.

## Conclusion

This study failed to provide any evidence on benefits of combined supplementation with curcumin and LCn-3PUFA in the current study. This could partly be due to uncertainties of co-administration of the two bio-actives, heterogeneity of the study population, gender balance in the groups or may be due to unknown interactions between these bio-actives. Further investigations are warranted on a single formulation with curcumin and LCn-3PUFA in a long-term trial to evaluate and elucidate the effects of curcumin and LCn-3PUFA on glycaemic control. In conclusion, curcumin supplementation has a favourable outcome on improving IS. On the other hand, LCn-3PUFA has shown profound effects on dyslipidaemia and AIP. Together, these results are indicative of a potential strategy for lowering the key risk factors in multifaceted progression of T2D.

## Additional file

Additional file 1: **Table S1.** Changes in the dietary intake, physical activity and fatty acid composition of the participants. **Table S2.** Mean changes in outcome measures stratified by sex within PL, CC, FO and CC-FO groups. **Figure S1.** Changes in the outcome measures from baseline to post intervention in people with fasting blood glucose levels (FBG) >5.5 and <5.5 in double placebo (PL), curcumin (CC), fish oil (FO) and curcumin + fish oil (CC-FO) groups; A. Change in the fasting blood glucose (%). B. Change in the serum insulin (%); C. Change in atherogenic index of plasma (AIP) (%) D. Change in HDL-Cholesterol (%). FBG >5.5: PL (*n*=4); CC (*n*=9); FO(*n*=10); CC-FO(*n*=12); FBG <5.5: PL (*n*=12); CC (*n*=6); FO(*n*=7); CC-FO(*n*=6). (DOCX 319 kb)

## Abbreviations

CC: Curcumin
CC-FO: Curcumin + fish oil
DHA: Docosahexaenoic acid
EPA: Eicosapentaenoic acid
FO: Fish oil
HbA1c: Glycosylated haemoglobin
HDL-C: High density Lipoprotein-Cholesterol
IFG: Impaired fasting glucose
IGT: Impaired glucose tolerance
IR: Insulin resistance
IS: Insulin sensitivity
LCn-3PUFA: Long chain omega-3 polyunsaturated fatty acids
PL: Placebo
T2D: Type 2 diabetes
TC: HDL-C
TC: Total cholesterol
TG: Triglycerides
Total: HDL-cholesterol ratio
