# Supplementary material for: Curcumin and/or omega-3 polyunsaturated fatty acids supplementation reduces insulin resistance and blood lipids in individuals with high risk of type 2 diabetes: a randomised controlled trial
Source: Lipids Health Dis. 2019 Jan 26;18:31. doi: 10.1186/s12944-019-0967-x (PMC6347796; doi:10.1186/s12944-019-0967-x)
Supplement: Supplementary file 1 — Table S1. Changes in the dietary intake, physical activity and fatty acid composition of the participants. Table S2. Mean changes in outcome measures stratified by sex within PL, CC, FO and CC-FO groups. Figure S1. Changes in the outcome measures from baseline to post intervention in people with fasting blood glucose levels (FBG) >5.5 and <5.5 in double placebo (PL), curcumin (CC), fish oil (FO) and curcumin + fish oil (CC-FO) groups; A. Change in the fasting blood glucose (%). B. Change in the serum insulin (%); C. Change in atherogenic index of plasma (AIP) (%) D. Change in HDL-Cholesterol (%). FBG >5.5: PL (n=4); CC (n=9); FO(n=10); CC-FO(n=12); FBG <5.5: PL (n=12); CC (n=6); FO(n=7); CC-FO(n=6). (DOCX 319 kb) [file 12944_2019_967_MOESM1_ESM.docx]

**Additional file 1: Table S1: Changes in the dietary intake, physical activity and fatty acid composition of the participants**

| CHARACTERISTICS | PL  (n=16) | CC  (n=15) | FO  (n=17) | CC-FO  (n=16) | P-VALUE |
| --- | --- | --- | --- | --- | --- |
| Dietary intake |  |  |  |  |  |
| Baseline (kj) | 8790 ± 632.85 | 9614 ± 534.9 | 8602 ± 317.3 | 8209 ± 432.7 | 0.257 |
| Change | 4 ± 467.0 | -327 ± 452.6 | -591 ±397.6 | 811 ± 446.6 | 0.131 |
| Physical activity |  |  |  |  |  |
| Baseline (METs-minutes/ week) | 3859 (5109) | 1692 (1597) | 2919 (4087.5) | 2970.5 (2718.2) | 0.257 |
| change | -349 (3103.3) | 61 (1508) | -539 (2012) | 113 (1120.2) | 0.409 |
| Erythrocyte fatty acid composition (%, w/w) |  |  |  |  |  |
| AA  Baseline | 15.6 (2.5) | 16 (2.2) | 15.5 (3.2) | 15.7 (1.6) | 0.148 |
| Change (%) | -1.9 (9.7) ^a^ | -2.7 (8.3) | -10.9 (10.3)** | -12.2 (7.0)**^a^ | 0.003 |
| EPA |  |  |  |  |  |
| Baseline | 0.96 (0.33) | 1.11 (0.77) | 1.05 (0.43) | 1.10 (0.36) | 0.067 |
| Change (%) | -3.02 (19.04)^b,c^ | -12.78 (4.07)* | 29.34 (59.47)**^b^ | 27.77 (61.88)**^c^ | 0.0001 |
| DHA |  |  |  |  |  |
| Baseline | 5.7 (1.4) | 6.5 (2.3) | 6.3 (2.4) | 6.4 (2.8) | 0.169 |
| Change (%) | 1.19 (16.4)^d,e^ | -3.69 (22.6) | 53.96 (43.6) **^d^ | 39.63 (56.0)**^e^ | 0.0001 |
| *n*-3 Index |  |  |  |  |  |
| Baseline | 6.63 (1.8) | 7.3 (2.3) | 7.6 (2.6) | 7.7 (3.3) | 0.190 |
| Change | 0.82 (16.38)^f,g^ | -4.16 (18.8) | 46.86 (44.53)**^f^ | 35.12 (56.69)**^g^ | 0.0001 |

Data is presented as mean ± SEM or median (IQR) as appropriate. Significant change from baseline, * p<0.05, ** p<0.01 represents the significant differences from the baseline with-in treatment groups. PL, placebo; CC, curcumin; FO, fish oil; CC-FO, curcumin + fish oil; AA, Arachidonic acid; EPA, Eicosapentaenoic acid; DHA, Docosahexaenoic acid; *n*-3 Index Omega-3 index. METs-minutes/week, metabolic equivalent minutes per week.

^a^ AA was significantly reduced in CC-FO (P=0.0035) group compared to the PL group. ^b^ EPA was significantly (P=0.0016) increased in the FO group compared to PL. ^c^EPA was significantly increased in CC-FO (P=0.0009) group compared to the PL. ^d^DHA was significantly increased in the FO (P=0.000007) group compared to PL. ^e^ DHA was significantly increased in the CC-FO (P=0.0018) group compared to PL. ^f^ *n*-3I was significantly increased in the FO (P=0.000006) group PL. ^g^*n*-3I was significantly increased in the CC-FO group compared to PL(P=0.0006).

**Additional file 1: Table S2: Mean changes in outcome measures stratified by sex within PL, CC, FO and CC-FO groups.**

|  | **PL** | | **CC** | | **FO** | | **CC-FO** | |
| --- | --- | --- | --- | --- | --- | --- | --- | --- |
| **Variable** | **Males**  **(n=7)** | **Females**  **(n=9)** | **Males**  **(n=6)** | **Females**  **(n=9)** | **Males**  **(n=7)** | **Females**  **(n=10)** | **Males**  **(n=6)** | **Females**  **(n=10)** |
| **Fasting glucose (%)** | -2.3 ± 1.8 | 0.7 ± 2.0 | -4.5 ± 4.1 | -0.1 ± 2.7 | -0.7 ± 3.4 | -0.4 ± 2.4 | 0.1 ± 2.2 | 0.7 ± 2.4 |
| **HbA1c (%)** | 2.0 ± 1.5 | 1.5 ± 0.7 | 0.4 ± 0.9 | 1.4 ± 1.0 | 1.3 ± 1.3 | 2.6 ± 0.9 | 3.9 ± 2.3 | 1.2 ± 1.3 |
| **Fasting insulin (%)** | 26.5 ± 29.6 | 10.1 ± 7.7 | -26.6 ±7.1 | -13.6 ± 7.6 | -10.2 ± 10.3 | -1.2 ± 9.5 | -9.6 ± 7.5 | 7.5 ± 12.4 |
| **HOMA2 IR** | 0.1 ± 0.2 | 0.1 ± 0.1 | -0.3 ± 0.1 | -0.2 ± 0.1 | -0.1 ± -0.1 | -0.1 ± 0.1 | -0.1 ± 0.1 | 0.0 ± 0.1 |
| **HOMA2 %S** | -5.4 ± 10.5 | -5.8 ± 6.2 | 45.9 ± 18.2 | 23.9 ± 0.3 | 20.3 ± 13.3 | 10.6 ± 11.4 | 15.6 ± 3.6 | 10.8 ± 10.6 |
| **InsuTAG** | 0.3 ± 0.2 | 0.3 ± 0.1 | -0.5 ± 0.2 | -0.2 ± 0.1 | -0.4 ± 0.1 | -0.2 ± 0.2 | -0.3 ± 0.1 | -0.0 ± 0.2 |
| **TG (%)** | 21.4 ± 8.6 | 31.2 ± 11.6 | -7.5 ± 15.1 | 3.8 ± 8.5 | -19.5 ± 6.6 | -14.3 ± 6.3 | -14.2 ± 9.3 | -0.6 ± 7.0 |
| **HDL-C (%)** | -3.0 ± 5.8 | -2.2 ± 2.2 | 6.2 ± 6.3 | 1.5 ± 3.4 | 10.7 ± 3.6 | 2.3 ± 5.2 | 8.6 ± 1.2 | 1.2 ± 7.9 |
| **AIP** | 0.2 ± 0.1 | 0.2 ± 0.1 | -0.1 ± 0.2 | -0.0 ± 0.1 | -0.3 ± 0.1 | -0.2 ± 0.1 | -0.3 ± 0.1 | -0.0 ± 0.1 |
| **TC (%)** | 0.6 ± 3.1 | -0.3 ± 1.1 | 5.5 ± 10.6 | 0.7 ± 3.7 | 3.2 ± 3.7 | 6.8 ± 6.2 | 7.6 ± 6.6 | 5.7 ± 4.1 |
| **LDL-C (%)** | -3.3 ± 2.7 | -4.3 ± 2.8 | 11.0 ± 17.6 | 0.4 ± 5.4 | 5.5 ± 3.8 | 11.6 ± 8.4 | 11.3 ± 7.3 | 8.4 ± 5.8 |

Data are presented as mean ± SEM. PL, placebo; CC, curcumin; FO, long chain omega-3 polyunsaturated fatty acids; CC-FO curcumin plus long chain omega-3 polyunsaturated fatty acids. HbA1c, Glycosylated haemoglobin; HOMA2 IR, Homeostatic model assessment for insulin resistance (log transformed); HOMA2 S, Homeostatic model assessment for insulin sensitivity; TG, Triglycerides; HDL-C high density lipoprotein-cholesterol; AIP, Atherogenic index of plasma; TC, total cholesterol, LDL-C, low density lipoprotein- cholesterol.

*

a

**Additional file 1: Figure S1:** Changes in the outcome measures from baseline to post intervention in people with fasting blood glucose levels (FBG) >5.5 and <5.5 in double placebo (PL), curcumin (CC), fish oil (FO) and curcumin + fish oil (CC-FO) groups; A. Change in the fasting blood glucose (%). B. Change in the serum insulin (%); C. Change in atherogenic index of plasma (AIP) (%) D. Change in HDL-Cholesterol (%). FBG >5.5: PL (n=4); CC (n=9); FO(n=10); CC-FO(n=12); FBG <5.5: PL (n=12); CC (n=6); FO(n=7); CC-FO(n=6)

Data presented as mean ± SEM or median (IQR) as appropriate. Significant changes from baseline indicated by * p<0.05. Means with same lower letter indicate significant differences –a. Fasting blood glucose levels were significantly lower in people with baseline FBG levels >5.5 with FO supplementation (P=0.008) compared to people with baseline FBG < 5.5.
